# Protein Thiol Modifications Visualized In Vivo

**DOI:** 10.1371/journal.pbio.0020333

**Published:** 2004-10-05

**Authors:** Lars I Leichert, Ursula Jakob

**Affiliations:** **1**Department of Molecular, Cellular, and Developmental Biology, University of MichiganAnn Arbor, MichiganUnited States of America

## Abstract

Thiol-disulfide interconversions play a crucial role in the chemistry of biological systems. They participate in the major systems that control the cellular redox potential and prevent oxidative damage. In addition, thiol-disulfide exchange reactions serve as molecular switches in a growing number of redox-regulated proteins. We developed a differential thiol-trapping technique combined with two-dimensional gel analysis, which in combination with genetic studies, allowed us to obtain a snapshot of the in vivo thiol status of cellular proteins. We determined the redox potential of protein thiols in vivo, identified and dissected the in vivo substrate proteins of the major cellular thiol-disulfide oxidoreductases, and discovered proteins that undergo thiol modifications during oxidative stress. Under normal growth conditions most cytosolic proteins had reduced cysteines, confirming existing dogmas. Among the few partly oxidized cytosolic proteins that we detected were proteins that are known to form disulfide bond intermediates transiently during their catalytic cycle (e.g., dihydrolipoyl transacetylase and lipoamide dehydrogenase). Most proteins with highly oxidized thiols were periplasmic proteins and were found to be in vivo substrates of the disulfide-bond-forming protein DsbA. We discovered a substantial number of redox-sensitive cytoplasmic proteins, whose thiol groups were significantly oxidized in strains lacking thioredoxin A. These included detoxifying enzymes as well as many metabolic enzymes with active-site cysteines that were not known to be substrates for thioredoxin. H_2_O_2_-induced oxidative stress resulted in the specific oxidation of thiols of proteins involved in detoxification of H_2_O_2_ and of enzymes of cofactor and amino acid biosynthesis pathways such as thiolperoxidase, GTP-cyclohydrolase I, and the cobalamin-independent methionine synthase MetE. Remarkably, a number of these proteins were previously or are now shown to be redox regulated.

## Abstract 2

A differential thiol-trapping technique combined with two- dimensional gel analysis has been developed and used to visualize thiol-disulfide exchange reactions, which act as switches in redox-regulated proteins

## Introduction

Cysteine is one of the most rarely used amino acids in the proteins of most organisms studied so far (Pe'er et al. 2004). Therefore, when highly conserved in proteins, it usually plays crucial roles in the structure, function, or regulation of the protein. This is due to the ability of thiol groups to stabilize protein structures by forming covalent disulfide bonds and to coordinate metal ions, as well as due to their high reactivity and redox properties.

Proteins in the extracellular space and oxidizing cell compartments (e.g., endoplasmic reticulum and periplasm) often rely on disulfide bonds to support their correct folding and maintain their structural stability (Bardwell 1994). Cytosolic proteins, on the other hand, are present within the reducing environment of the cytosol. Here, cysteine residues are reduced and often found in binding pockets of substrates, coenzymes, or metal cofactors (e.g., in zinc binding dehydrogenases), or are present in the active site of enzymes, where they directly participate in the catalytic reaction (e.g., in cysteine proteases). Moreover, cysteine residues are also often involved in redox reactions, where transfer of electrons proceeds via thiol-disulfide exchange reactions. Importantly, the activity of all these cytosolic enzymes usually depends on the preservation of the reduced state of the cysteine residue(s) involved.

The very same properties that make cysteine the perfect amino acid for redox reactions, metal coordination and thiol-disulfide interchanges, also make cysteines extremely vulnerable to oxidation by reactive oxygen species (ROS). ROS arise transiently during normal metabolism as toxic byproducts of respiration and have been shown to accumulate under conditions of oxidative stress. Over the past few years, an increasing number of thiol-containing proteins has been identified that use ROS as a mediator to quickly regulate their protein activity (Linke and Jakob 2003). This new class of redox-regulated proteins includes the molecular chaperone Hsp33, which we discovered in 1999 (Jakob et al. 1999), metabolic enzymes (e.g., glyceraldehyde-3-phosphate dehydrogenase [GapDH]) (Cotgreave et al. 2002), prokaryotic and eukaryotic transcription factors (OxyR and Yap1) (Rainwater et al. 1995; Zheng et al. 1998; Kang et al. 1999; Kuge et al. 2001; Kim et al. 2002), kinases (protein kinase C and Raf) (Gopalakrishna and Jaken 2000; Hoyos et al. 2002), and phosphatases (PTP1B and PTEN) (Barrett et al. 1999; Leslie et al. 2003). What all these proteins have in common are highly reactive cysteine residues that are quickly and reversibly modified upon exposure to oxidative stress. These modifications include disulfide bond formation (e.g., in Hsp33, RsrA, and OxyR), nitrosylation (e.g., in Ras and OxyR), glutathionylation (e.g., in PTP1B, GapDH, and OxyR), or sulfenic acid formation (e.g., in PTP1B and OxyR). These modifications cause significant conformational changes and either lead to the activation (e.g., in Hsp33, OxyR, PKC, and Raf-kinase) or inactivation (e.g., in p53 and PTEN) of the respective protein's function. Upon return to non–oxidative stress conditions, cellular reductants such as the small molecule glutathione as well as cellular reductases like thioredoxin and glutaredoxin rapidly reduce the cysteine modifications and restore the original protein activity. These findings suggested that basically any protein with reactive cysteine residue(s) has the potential of being redox regulated. Many important regulatory proteins such as zinc finger proteins contain clusters of conserved cysteines and are, therefore, attractive targets for redox regulation.

Over the past few years, several proteomic strategies have been developed to identify proteins that undergo thiol modifications in vivo. These methods addressed very specific questions and were used to either identify disulfide-bonded or glutathionylated proteins under oxidative stress conditions in vivo (Fratelli et al. 2002; Cumming et al. 2004), thioredoxin-targeted proteins in chloroplasts and Escherichia coli (Motohashi et al. 2001; Yano et al. 2001; Kumar et al. 2004), or target proteins of periplasmic thiol-disulfide oxidoreductases (Hiniker and Bardwell 2004; Kadokura et al. 2004). None of these methods, however, generated a general and global overview of thiol-modified proteins in vivo. We have now invented a differential thiol-trapping technique combined with two-dimensional (2D) gel analysis to monitor the in vivo thiol status of cellular proteins upon variations in the redox homeostasis of the cells. To test our method, we analyzed the thiol-disulfide status of proteins in aerobically growing E. coli cells and confirmed that the majority of proteins with thiol modifications are localized to the oxidizing environment of the periplasm. We found that the periplasmic thiol-disulfide oxidoreductase DsbA is responsible for these protein thiol modifications and identified novel DsbA substrate proteins. We then used our method to visualize directly the extent of ROS-induced thiol oxidation during aerobic growth and identified a number of cytosolic proteins with reactive cysteine residues that require functional thioredoxin to maintain their reduced thiol state. Finally, we analyzed the thiol status of cellular proteins in cells that were exposed to H_2_O_2_-induced oxidative stress and discovered a select group of new potentially redox-regulated proteins in vivo.

## Results

### A Differential Trapping Technique to Detect Oxidatively Modified Proteins In Vivo

**Figure 1:**
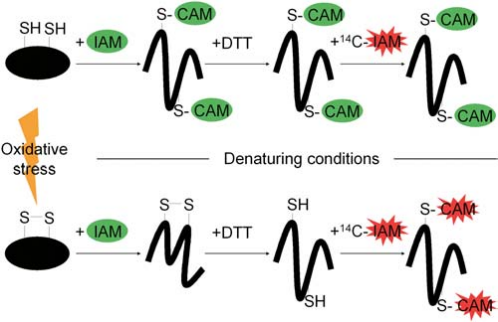
Schematic Overview of Our Differential Thiol-Trapping Technique Under normal growth conditions (top), a hypothetical cytoplasmic protein present within the complex mixture of the crude whole-cell extract is fully reduced. Upon incubation in TCA, all thiol-disulfide exchange reactions are quenched and the cells lyse. In the first thiol-trapping step, the protein is denatured and incubated with IAM. Accessible thiol groups are quickly carbamidomethylated (CAM) and blocked for the subsequent reduction/alkylation steps. After TCA precipitation and washing, DTT is added to reduce oxidized cysteines, and ^14^C-labeled IAM is used to modify potentially newly released, accessible cysteines. Under oxidative stress conditions (bottom) the cysteine residues become modified (e.g., sulfenic acid and disulfide bonds). In the first trapping step, IAM cannot attack the oxidized disulfide bond. Only after reduction with DTT are the cysteines accessible to the ^14^C-labeled IAM. Therefore, the ^14^C radioactivity correlates with the degree of thiol modification in the protein. The differentially trapped protein species are chemically identical regardless of their original thiol-disulfide status. This ensures their identical migration behavior on 2D gel electrophoresis.

We have developed an innovative technique that allows us to monitor globally the in vivo thiol status of cellular proteins upon variations in the redox homeostasis of the cells. This method is based on the sequential reaction of two variants of the thiol-modifying reagent iodoacetamide (IAM) with accessible cysteine residues in proteins (Figure 1). Wild-type or mutant cells that were grown exponentially in glucose-minimal medium at 37 ^o^C were exposed to the desired oxidative stress treatment. Then, cells were treated with trichloracetic acid (TCA) to rapidly quench thiol-disulfide exchange reactions. All accessible thiol groups were then alkylated with cold, unlabeled IAM under denaturating conditions. In a next step, all reversible thiol modifications that developed during normal growth or oxidative stress treatment (e.g., disulfide bonds and sulfenic acids) were reduced with DTT, and the newly accessible thiol groups were modified with ^14^C-labeled IAM. Therefore, radioactivity was specifically incorporated into proteins that originally contained thiol modifications. High ratios of ^14^C activity/protein are predicted for proteins with thiol modifications while low ratios of ^14^C activity/protein are predicted for proteins whose thiol groups are not significantly modified in vivo (Figure 1).

The differentially trapped protein extract was then separated by 2D gel electrophoresis. Importantly, due to this specific trapping technique, all accessible thiol groups of each protein were carbamidomethylated to an extent that is independent of the original thiol-disulfide status of the protein. This ensures their identical migration behavior on 2D gels. The 2D gels were stained with colloidal Coomassie blue to get a measure of the total protein content. The ^14^C radioactivity, which correlates to the degree of thiol modification in the individual spots, was determined by exposing the dried gels to phosphor screens. Then, the ^14^C activity/protein ratio was visualized and quantified.

### The Majority of Oxidized Proteins Are Present in the Periplasm of E. coli

**Figure 2:**
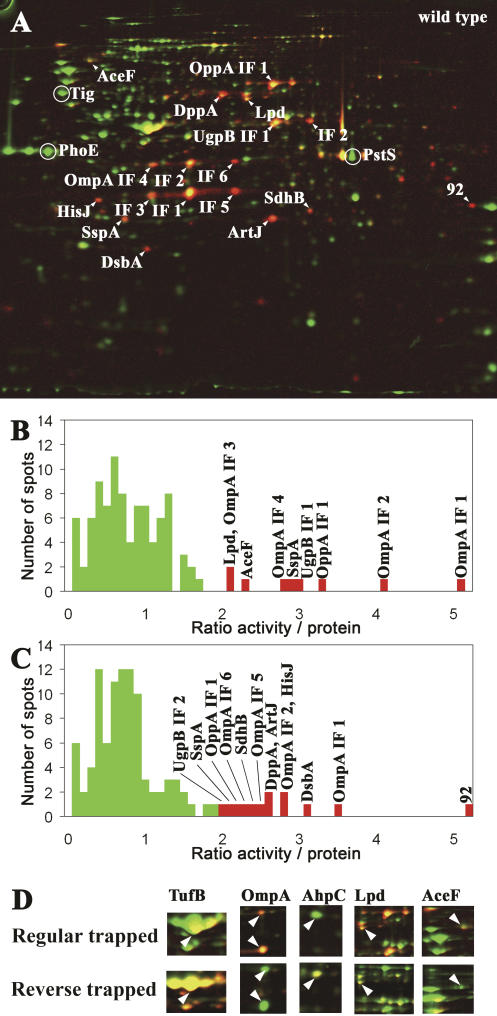
Overall Thiol-Disulfide State of Cellular Proteins in Exponentially Growing E. coli Wild-Type Cells (A) Colored overlay of the Coomassie blue–stained 2D gel (shown in green) and the phosphor image (shown in red) of a differentially trapped protein extract from exponentially growing E. coli wild-type cells. Proteins with a high ratio of ^14^C activity/protein appear red; proteins with a low ratio appear green. Protein spots with a ratio of ^14^C activity/protein greater than 2.0 are indicated by an arrow, while circles label abundant proteins without cysteines. (B) Distribution of the ^14^C activity/protein ratio in the 100 most abundant protein spots found on a Coomassie blue–stained gel. Bars representing spots with a ratio higher than 2.0 are colored red and are labeled with the name of the protein(s) they represent. (C) Distribution of the ^14^C activity/protein ratio in the 100 most intense protein spots found on the phosphor images. Bars representing spots with a ratio higher than 2.0 are colored red and are labeled with the name of the protein(s) they represent. (D) Regular and reverse trapping of exponentially growing E. coli wild-type cells. Details of colored overlays of stained protein gels (shown in green) and phosphor images (shown in red) of cell extracts upon regular trapping (top) and reverse trapping (bottom).

In order to test our method, we analyzed the steady-state thiol-disulfide status of cellular proteins in wild-type *E. coli. E. coli* strains were grown in minimal medium to mid-logarithmic phase, and the cells were harvested. The cysteines were thiol trapped using our differential thiol-trapping technique and separated on 2D gels. To analyze the extent of thiol modification and the distribution of thiol-modified proteins in an unbiased way, we focused first on the 100 most abundant proteins on our colloidal Coomassie blue–stained 2D gels. We set the total spot intensity of all 100 protein spots on the colloidal blue gel to 100% and determined the relative spot intensity for each protein. We then quantified the 100 corresponding ^14^C activity spots on the phosphor image, set their combined total spot intensities to 100%, and determined again the relative spot intensity for each protein. Finally, we determined the ^14^C activity/protein ratios for each of the 100 proteins. Low ^14^C activity/protein ratios were predicted for non-thiol-modified proteins presumably present in the reducing environment of the cytoplasm, while high ratios of ^14^C activity/protein were predicted for proteins with cellular thiol modifications such as those present in the oxidizing milieu of the E. coli periplasm. As shown in Figure 2, the majority of proteins (91 proteins) had a ^14^C activity/protein ratio below 2.0, while nine proteins showed a higher than 2.0-fold ratio.

Mass spectrometric identification of a large number of these proteins suggested that our differential thiol trapping is indeed very selective for proteins with thiol modifications. From the nine protein spots with a ^14^C activity/protein ratio greater than 2.0, six are known periplasmic proteins such as the periplasmic oligopeptide permease (OppA) and glycerol-uptake protein (UgpB ), as well as proteins associated with the outer membrane like the outer membrane porin protein A (OmpA) (Figure 2A and 2B). Importantly, all of these proteins harbor at least two cysteines, suggesting that they may form structural disulfide bonds in the oxidizing environment of the periplasm.

Three potentially cytoplasmic proteins were found to have high ^14^C activity/protein ratios: dihydrolipoyl transacetylase (AceF), lipoamide dehydrogenase (Lpd), and the stringent starvation protein (SspA) (Figure 2A and 2B). AceF and Lpd correspond to the enzymes E2 and E3 of the pyruvate dehydrogenase complex. Lpd contains a reactive cysteine pair in the active site that undergoes disulfide bond formation during the regeneration of the disulfide bond of the covalently bound cofactor lipoamide of AceF (Massey and Veeger 1960). Detection of these proteins in our analysis is an excellent indication that we obtained an in vivo snapshot of proteins that use disulfide bond formation in their catalytic cycle and shows that the method can detect the redox state of covalently bound thiol-containing cofactors as well. SspA contains only one cysteine residue. This cysteine residue might be glutathionylated in vivo under steady-state conditions. Alternatively, however, SspA might co-migrate with the low-abundance periplasmic protein arginine binding protein ArtI that harbors two highly conserved cysteines and migrates at a very similar position on periplasmic extract gels (A. Hiniker, personal communication).

The majority of cytoplasmic proteins that contain numerous cysteines, on the other hand, revealed a ^14^C activity/protein ratio below 2.0, including the very abundant elongation factor EF-Tu (Tu elongation factor [TufB]-IF1) with three cysteine residues (^14^C activity/protein ratio = 1.3 ± 0.5) and isocitrate dehydrogenase with seven cysteine residues (^14^C activity/protein = 1.2 ± 0.5). Protein spots that showed extremely low ^14^C activity/protein ratios (less than 0.2) included the very abundant outer membrane porin protein E as well as the cytoplasmic proteins P-specific transport protein and trigger factor , all of which do not contain any cysteine residues (Figure 2A). These results indicated that under our labeling conditions, IAM was quite specific for cysteine residues, and labeling of non-thiol-containing amino acids could be neglected.

Because many proteins with thiol modifications are low-abundance periplasmic proteins, we performed a similar analysis but focused now on the 100 most heavily ^14^C-modified proteins rather than on the 100 most abundant proteins (Figure 2C). A number of proteins that showed a high ^14^C activity/protein ratio were periplasmic proteins that we had previously identified. In addition, however, the low-abundance periplasmic proteins periplasmic histidine binding protein (HisJ), ArtJ, DsbA, and the periplasmic dipeptide binding protein (DppA) were identified as proteins with a very high degree of thiol modification (^14^C activity/protein ratio > 2.0) (Figure 2A and 2C). All four proteins are known to be localized to the periplasm of E. coli and again contain at least one pair of cysteine residues. Both DppA and HisJ have recently been identified as substrates of the disulfide oxidase DsbA (Hiniker and Bardwell 2004), confirming that they do contain disulfide bonds in vivo.

To obtain an idea about the sensitivity of our method, we closely analyzed DsbA, a protein that contains two cysteine residues and that has been found to be fully oxidized in wild-type E. coli cells using our technique as well as conventional thiol-trapping methods (Kishigami et al. 1995). Although DsbA was only a faint spot on protein gels (318 spots showed a stronger signal), it was among the most abundant spots on the phosphor image (the 27th most intense spot). This showed that even in a low-abundant protein such as DsbA, the presence of only two thiol-modified cysteines is fully sufficient to create a clearly detectable ^14^C signal.

### Regular and Reverse Thiol Trapping—Determining Redox States In Situ

Quantitative analysis of the ratio of oxidized and reduced protein species in vivo can be used to determine the redox state of the protein in the cell providing that the oxidation mechanism of the protein is known (Watson and Jones 2003). We therefore considered that if our regular thiol trapping was completely alkylating all reactive cysteine residues, the ^14^C activity/protein ratio of a defined protein spot should correspond to the amount of oxidized protein. To then visualize and quantify the amount of reduced protein species in the same protein spot, we decided to perform a reverse trapping in parallel. In the reverse trapping, all free, accessible cysteines were immediately alkylated with radioactive IAM, while oxidatively modified cysteines were alkylated with cold IAM after their reduction. Therefore, the ^14^C activity/protein ratio should now correspond to the amount of reduced protein in the respective protein spot. We confirmed that proteins that had very high ratios of ^14^C activity/protein such as oxidized OmpA IF1 and IF2 in our regular trapping had very low ratios of ^14^C activity/protein (0.2 ± 0.08) in our reverse-trapped samples (Figure 2D). A mostly reduced protein such as succinyl-CoA synthetase, which had a low ratio of 0.7 ± 0.2 under regular trapping conditions, on the other hand showed a very high ratio of 2.8 ± 0.7 under reverse-trapping conditions. Based on these results, we considered that the comparison of the ^14^C activity/protein ratio of defined protein spots in regular and reverse-trapped samples could give us the ratio of oxidized and reduced protein under steady-state conditions, if the reaction mechanism of the protein oxidation was known.



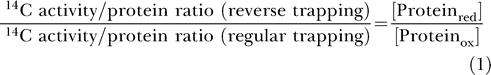



This should then allow us to determine the half-cell potential or redox state of the cellular proteins in vivo. To test our approach, we decided to calculate the redox state of Lpd, a cytosolic enzyme that we identified in our screen to be partly oxidized under steady-state conditions. Oxidative decarboxylation of pyruvate goes along with the reduction of lipoamide, the prosthetic group of AceF. To regenerate this complex, the disulfide bond in dihydrolipoamide is re-oxidized by the active-site disulfide bond of Lpd, which itself donates its electrons to the prosthetic flavin adenine dinucleotide and ultimately to nicotinamide adenine dinucleotide. Because the standard redox potentials of Lpd and the dihydrolipoic acid/lipoic acid redox pair of AceF are known (Table 1) (Maeda-Yorita et al. 1991; Nelson et al. 2000), we determined the ^14^C activity/protein ratio of Lpd under regular and reverse-trapping conditions and determined its redox state in vivo.

**Table 1:**
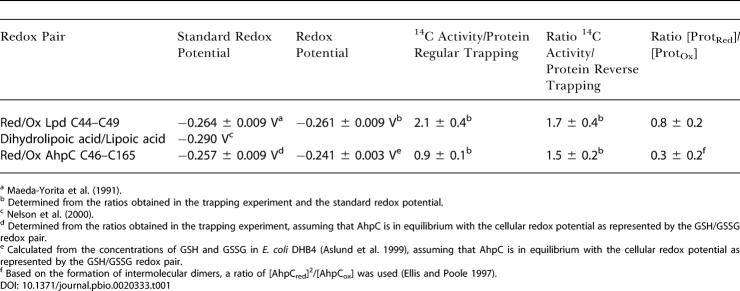
Standard Redox Potentials, Redox Potentials, and ^14^C Activity/Protein Ratios in the Reverse and Regular Trapping Experiments of Selected Redox Pairs

^a^ Maeda-Yorita et al. (1991)

^b^ Determined from the ratios obtained in the trapping experiment and the standard redox potential

^c^ Nelson et al. (2000)

^d^ Determined from the ratios obtained in the trapping experiment, assuming that AhpC is in equilibrium with the cellular redox potential as represented by the GSH/GSSG redox pair

^e^ Calculated from the concentrations of GSH and GSSG in E. coli DHB4 (Aslund et al. 1999), assuming that AhpC is in equilibrium with the cellular redox potential as represented by the GSH/GSSG redox pair

^f^ Based on the formation of intermolecular dimers, a ratio of [AhpC_red_]^2^/[AhpC_ox_] was used (Ellis and Poole 1997)



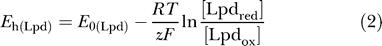



The in vivo redox state of Lpd was found to be –0.261 ± 0.009 V (pH 7.0). This was in excellent agreement with the proposed flow of electrons through this multienzyme system, showing that Lpd is able to oxidize the dihydrolipoic acid in AceF (*E*_0_ = −0.290). A reliable direct measurement of the redox potential of the dihydrolipoic acid/lipoic acid redox couple in AceF was not possible because of the very low signal that we found for AceF in the reverse-trapping experiment. This indicated that AceF is mostly in its thiol-oxidized state. This probably reflects the fact that the prosthetic group is only accessible to Lpd in its reduced state (Nelson et al. 2000), which presumably allows AceF to keep its prosthetic group oxidized even within the very reducing environment of the cytosol. The redox potential that we determined for Lpd also suggested that the components of this multienzyme complex are neither in equilibrium with one another nor with the overall GSH/GSSG redox potential in the cell (−0.24 V at pH 7.0, an intracellular concentration of 5 mM GSH, and a ratio of GSH/GSSG of 223:1 in E. coli DHB4 [Aslund et al. 1999]). These results showed that the calculation of (standard) redox potentials for proteins in vivo using our differential trapping technique was possible when sufficient amounts of reduced and oxidized species could be detected and when their ratios could be reliably quantified. Then, this technique proved to be very useful to estimate the direction of electron flow in metabolic pathways in vivo.

We also calculated the standard redox potential of alkylhydroperoxide reductase small subunit (AhpC), a protein that uses disulfide bond formation to detoxify alkylhydroperoxides, assuming that under steady-state conditions it is in equilibrium with the overall cellular redox potential. We calculated a standard redox potential for AhpC of −0.257 ± 0.009 V. This agrees well with studies in Helicobacter pylori (Baker et al. 2001) and our findings (see below) that suggested that thioredoxin (*E*_0_ = −0.270 V) (Krause et al. 1991) might play a direct role in the catalytic cycle of AhpC.

### Identification of the In Vivo Substrate Proteins of DsbA

We found that our method specifically and reliably detected proteins with thiol modifications in vivo. This suggested to us that our method should also be an excellent tool to determine the in vivo substrate specificity of cellular thiol-disulfide oxidoreductases. We therefore decided to first compare the thiol-disulfide status of proteins in wild-type E. coli and strains that lack DsbA, the enzyme that is responsible for disulfide bond formation in the periplasm of E. coli. Previously, only a few DsbA substrates have been identified. The studies that addressed this question in the past relied either on the formation of covalent intermediates between an active-site cysteine mutant of DsbA and substrate proteins (Kadokura et al. 2004), or on the instability and premature degradation of periplasmic proteins that are no longer stabilized by disulfide bonds because of the absence of DsbA (Hiniker and Bardwell 2004).

**Figure 3:**
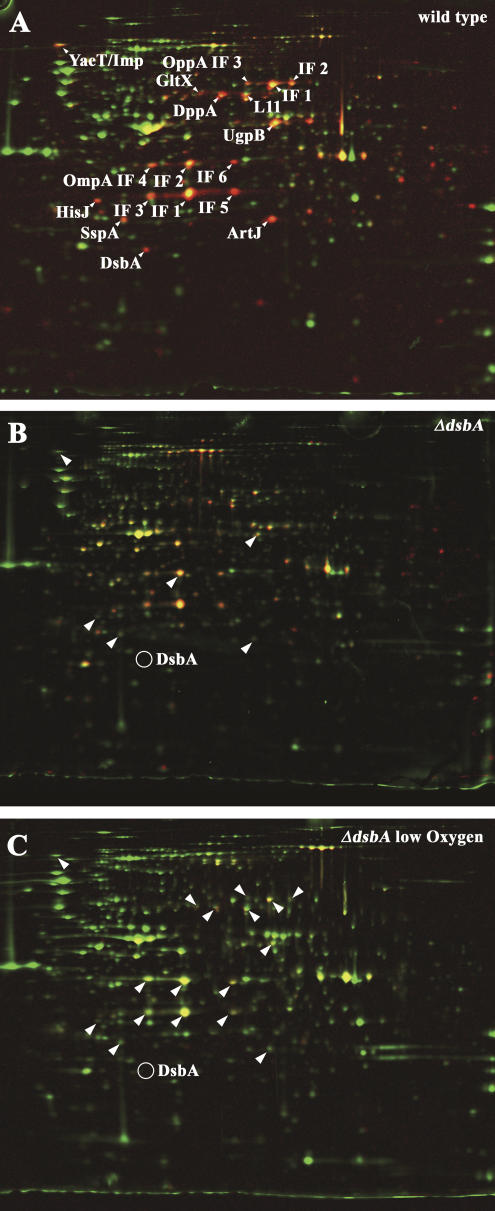
Identification of In Vivo Substrate Proteins of the Periplasmic Disulfide Bond Oxidase DsbA (A) Colored overlay of the stained 2D gel (shown in green) and the phosphor image (shown in red) of differentially trapped protein extract from exponentially growing E. coli wild-type cells. Proteins with a high ^14^C activity/protein ratio appear red, while proteins with a low ratio appear green. Proteins that were found to have significantly lower ratio of ^14^C activity/protein in the *dsbA*::kan strain (B and C) are labeled. (B) Overlay of the stained 2D gel (shown in green) and the phosphor image (shown in red) of a differentially trapped protein extract from exponentially growing *dsbA*::kan cells. Proteins that were found to have a significantly lower ^14^C activity/protein ratio in *dsbA*::kan cells than in wild-type strain (A) are marked with an arrowhead. A circle marks the position of DsbA on the wild-type gel. (C) Overlay of the stained 2D gel (shown in green) and the phosphor image (shown in red) of a differentially trapped protein extract from *E. coli dsbA*::kan cells growing under oxygen limitation. Arrowheads label proteins that were found to have a significantly lower ^14^C activity/protein ratio than the wild-type strain grown under oxygen limitation. A circle marks the position of DsbA on the wild-type gel.

We grew wild-type E. coli cells and cells lacking the chromosomal copy of DsbA (*dsbA::*kan) to mid-logarithmic phase, harvested the cells, and differentially thiol-trapped the cysteines. As shown in Figure 3 and Table 2, using our new thiol-trapping technique, we identified a number of proteins that showed significantly less or no thiol modification in *dsbA* deletion strains than in wild-type strains. Among the proteins that we selected for mass spectrometric analysis were known DsbA substrate proteins (e.g., OmpA, DppA, organic solvent tolerance protein [Imp], and HisJ) as well as a number of proteins that have not yet been associated with DsbA (e.g., ArtJ and UgpB). Because all of these proteins are periplasmic and have at least one conserved pair of cysteines, it appears very likely that these proteins are also substrate proteins of DsbA.

**Table 2:**
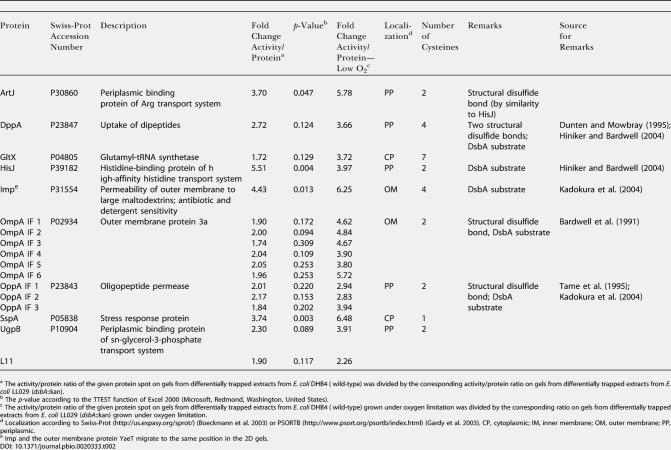
Identification of the In Vivo Substrates of DsbA

^a^ The activity/protein ratio of the given protein spot on gels from differentially trapped extracts from E. coli DHB4 ( wild-type) was divided by the corresponding activity/protein ratio on gels from differentially trapped extracts from E. coli LL029 (*dsbA*::kan)

^b^ The *p*-value according to the TTEST function of Excel 2000 (Microsoft, Redmond, Washington, United States)

^c^ The activity/protein ratio of the given protein spot on gels from differentially trapped extracts from E. coli DHB4 ( wild-type) grown under oxygen limitation was divided by the corresponding ratio on gels from differentially trapped extracts from E. coli LL029 (*dsbA*::kan) grown under oxygen limitation

^d^ Localization according to Swiss-Prot (http://us.expasy.org/sprot/) (Boeckmann et al. 2003) or PSORTB (http://www.psort.org/psortb/index.html) (Gardy et al. 2003). CP, cytoplasmic; IM, inner membrane; OM, outer membrane; PP, periplasmic

^e^ Imp and the outer membrane protein YaeT migrate to the same position in the 2D gels

Interestingly, the oxidation state of the known DsbA substrate protein OmpA, as well as of some other periplasmic proteins, appeared to be only moderately affected by the lack of DsbA (Figure 3A and 3B). Because the process of folding and disulfide bond formation in OmpA has been shown to be reasonably fast (half time, *t*_1/2_ = 5 min) even in the absence of DsbA (Bardwell et al. 1991), we considered that air oxidation was probably responsible for the disulfide bond formation in proteins such as OmpA under steady-state conditions. To investigate the role that air oxidation might play in the disulfide bond formation of periplasmic proteins in vivo, we performed the same thiol-trapping experiments using wild-type and *dsbA*::kan cells that were grown under very limiting oxygen conditions. Under those oxygen-limited conditions the ^14^C activity/protein ratio of OmpA and other periplasmic proteins was dramatically decreased compared to wild-type cells and also significantly lower than in *dsbA*::kan cells grown under normal oxygen conditions (Figure 3C; Table 2). These results not only confirmed that the thiol modifications that we detected with our differential thiol-trapping technique are indeed in vivo modifications and are not introduced during the aerobic lysis and trapping of the sample, but also clearly showed that DsbA is not absolutely necessary for the thiol oxidation of certain periplasmic proteins under aerobic conditions. Under low-oxygen conditions, however, which occur in stationary phase or when cells are grown micro-aerobically, functional DsbA is absolutely required for the successful disulfide bond formation in the E. coli periplasm.

### TrxA Protects a Large Number of Intracellular Proteins from Oxidation

While DsbA promotes disulfide bond formation in the E. coli periplasm, the thioredoxin and glutaredoxin systems reduce disulfide bonds in the E. coli cytoplasm. These systems not only prevent the formation of unwanted disulfide bonds in cytoplasmic proteins, which often lead to the inactivation of the respective proteins, but also play important regulatory roles in the cell. For instance, the oxidative stress response in both prokaryotes and eukaryotes is rapidly attenuated by glutaredoxins and thioredoxins, which reduce and inactivate the oxidative stress transcription factors OxyR or Yap1p (Carmel-Harel and Storz 2000). Therefore, analysis of the proteins that use these systems for their specific reduction will help us to identify cytosolic proteins that use thiol modifications in their functional life cycle.

**Figure 4:**
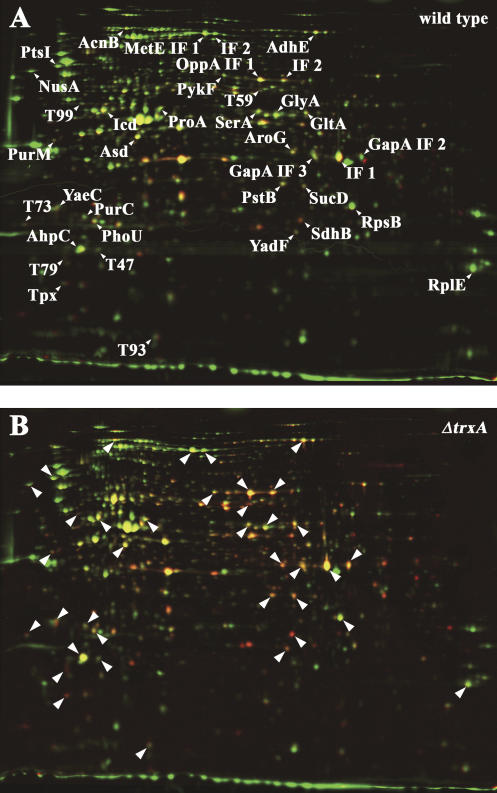
Identification of the In Vivo Substrate Proteins of Thioredoxin A (A) Colored overlay of the stained 2D gel (shown in green) and the phosphor image (shown in red) of differentially trapped protein extract from exponentially growing E. coli wild-type cells. Proteins with a high ratio of activity per protein appear red, proteins with a low ratio appear green. Proteins that were found to have a significantly higher ratio of activity per protein in the *trxA^−^* strain (B) are labeled. (B) Overlay of the stained 2D gel (shown in green) and the phosphor image (shown in red) of differentially trapped protein extract from exponentially growing E. coli Δ*trxA* cells. Proteins that were found to have a significantly higher ratio of ^14^C activity/protein in the Δ*trxA* strain are labeled with an arrow.

To analyze the substrate specificity of cytoplasmic thiol-disulfide oxidoreductases, we decided to compare the thiol-disulfide status of proteins in a thioredoxin null mutant and the isogenic wild-type E. coli strain. Importantly, strains that lack the *trxA* gene do not exhibit a general disulfide stress phenotype (Prinz et al. 1997), which minimizes potential secondary thiol modifications in proteins that could be otherwise attributed to those stresses (Derman et al. 1993). The dramatic alteration in the oxidation state of a large number of cellular proteins in a Δ*trxA* strain as compared to a wild-type E. coli strain is clearly visible (Figure 4). Of the 100 proteins that were selected based on their high level in ^14^C activity, 37 protein spots showed a more than 2-fold further increase in thiol modification in Δ*trxA* strains compared to wild-type strains, where functional thioredoxin is apparently working successfully to keep them reduced. Proteins whose ^14^C activity/protein ratio did not change in the absence of thioredoxin included the majority of periplasmic proteins that have been identified before, as well as some highly abundant cytoplasmic proteins that harbor presumably inaccessible or unreactive cysteines.

All 37 spots were selected for mass spectrometric analysis, and resulted in the identification of 27 individual proteins, 23 of which have either been shown or are predicted to be localized to the reducing environment of the cytoplasm and to contain at least one and up to ten cysteine residues (Table 3). Of those, at least six proteins (e.g., thioredoxin-linked thiol peroxidase [Tpx], AhpC, GapDH, and aconitase B [AcnB]) have been previously shown to be targets of thioredoxin in plants (Yamazaki et al. 2004) or, very recently, in E. coli (Kumar et al. 2004) (Table 3). Thirteen proteins have cysteine residues in the active site or the cofactor-binding site (e.g., aspartate semialdehyde dehydrogenase, citrate synthase, and γ-glutamyl phosphate reductase). Five proteins are known metal-binding proteins either coordinating zinc via cysteines (e.g., cobalamin-independent methionine synthase [MetE] and carbonic anhydrase [YadF]), binding iron (e.g., alcohol/acetaldehyde dehydrogenase[AdhE]), or harboring iron-sulfur clusters (e.g., AcnB and succinate dehydrogenase [SdhB]) (Table 3). Reactive and/or accessible cysteine residues appear to make these proteins particularly vulnerable to small amounts of ROS, such as hydrogen peroxide (H_2_O_2_), which are known to be produced as toxic byproducts of cellular respiration in aerobically growing cells (Costa Seaver and Imlay 2001). That H_2_O_2_ must be produced during aerobic growth became also obvious when we found the detoxifying enzymes AhpC and the thioredoxin-linked Tpx to be largely oxidized. Both proteins use disulfide bond formation to detoxify peroxides and appear to require functional thioredoxin to regenerate their reduced thiol status.

**Table 3:**
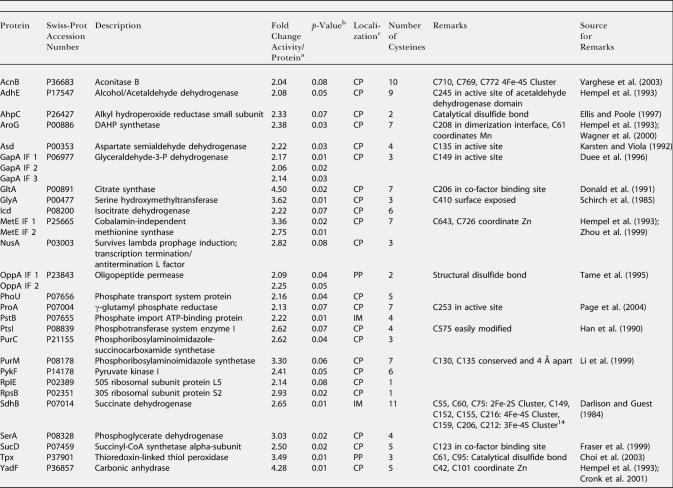
In Vivo Substrate Proteins of Thioredoxin A

^a^ The ^14^C activity/protein ratio of the given protein spot on gels from differentially trapped extracts from E. coli DHB4 ( wild-type) was divided by the corresponding ^14^C activity/protein ratio on gels from differentially trapped extracts from E. coli WP570 (Δ*trxA*)

^b^ The *p*-value according to the TTEST function of Excel 2000

^c^ Localization according to Swiss-Prot (http://us.expasy.org/sprot/) (Boeckmann et al. 2003) or PSORTB (http://www.psort.org/psortb/index.html) (Gardy et al. 2003). CP, cytoplasmic; IM, inner membrane; OM, outer membrane; PP, periplasmic

**Table 3:**
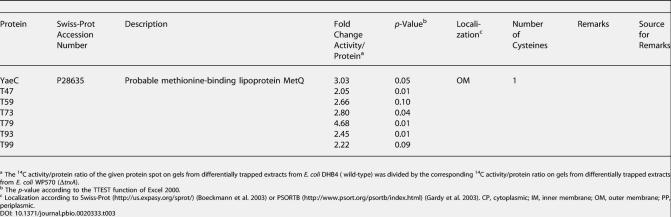
Continued

The in vivo snapshot of the thiol status of proteins in thioredoxin-defective strains shows for the first time, to our knowledge, the detrimental effects of small amounts of ROS generated during aerobic growth on cytosolic proteins, and the important role that thioredoxin A plays in regenerating these proteins. A surprisingly large number of cytosolic proteins appear to harbor such oxidation-sensitive cysteine residues that require the constant presence of the reducing thioredoxin system under aerobic growth. We cannot exclude at this point the possibility that the other cellular redox system glutaredoxin is overwhelmed by the accumulation of thiol-modified proteins in thioredoxin-deficient strains as well. This might be the reason why we detect thiol-modified MetE, one of the very few known glutathionylated E. coli proteins (Hondorp and Matthews 2004), which presumably requires functional glutaredoxin for its regeneration. It is also important to note that we focused only on the 100 most intense protein spots on the phosphor image. A significant number of additional proteins showed an at least 2-fold further increase in their ^14^C activity/protein ratio in *trxA^−^* cells as compared to wild-type cells. These are likely also substrates of thioredoxin and remain to be identified (Figure 4).

Only three periplasmic proteins were found to be increasingly thiol-modified in the absence of cytoplasmic thioredoxin; Tpx, OppA , and the methionine-binding protein MetQ (YaeC). In the case of Tpx, thioredoxin A has been suggested to be an essential component in its functional regulation. This makes Tpx a potential substrate protein of the periplasmic thiol-disulfide oxidoreductase systems DsbC or DsbG, which are connected to cytoplasmic thioredoxin via the membrane protein DsbD. Absence of thioredoxin in the cytoplasm would lead to the accumulation of oxidized DsbC and DsbG in the periplasm, which would then no longer be able to reduce and/or isomerize disulfide bonds in periplasmic proteins such as Tpx; this would explain the accumulation of oxidized proteins in the periplasm. The same could apply for the other two periplasmic proteins that we identified in this study. Alternatively, however, thiol modifications that occur and are not reduced in the cytoplasm might prevent their efficient transport, or might impair potential attachments of lipid anchors. The latter might be the case for the lipid protein MetQ (YaeC), whose single cysteine is predicted to be linked to a lipid anchor.

### Identification of Proteins Sensitive to Oxidative Stress

Over the past few years, an increasing number of redox-sensitive proteins have been identified that use the oxidation state of reactive cysteine residues as a regulatory switch. Oxidative stress–induced thiol modifications lead to conformational changes and to the transient activation or inactivation of the respective protein. Upon return to non–oxidative stress conditions, cellular reductants such as the thioredoxin system rapidly reduce the cysteine modifications and restore the original protein activity. The observation that the function of so many different cysteine-containing proteins is regulated by the redox conditions of the environment, suggests that basically any protein with one or more reactive and exposed cysteines has the potential of being redox-regulated. Because many important regulatory and biosynthetic proteins contain clusters of cysteines in the active site or cofactor-binding site, they can therefore be considered attractive and potential targets for this novel form of functional regulation.

**Figure 5:**
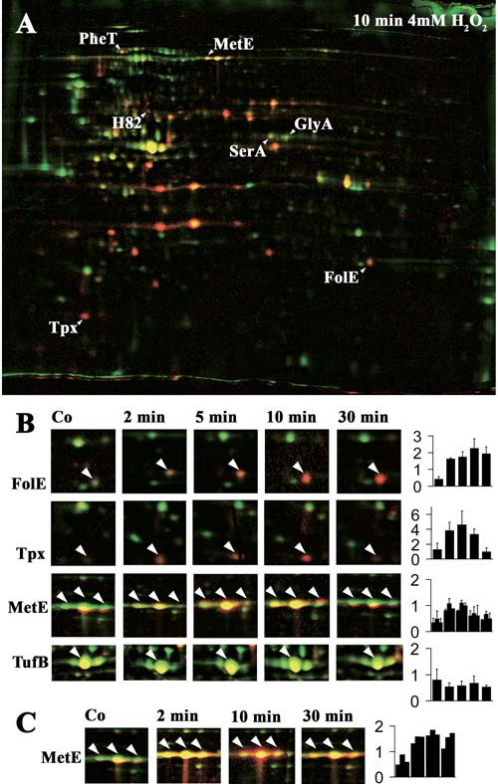
Identification of Oxidative Stress–Sensitive Proteins In Vivo (A) Overlay of the stained 2D gel (shown in green) and the phosphor image (shown in red) of differentially trapped protein extracts from H_2_O_2_-stressed E. coli wild-type cells. Proteins that were found to have a significantly higher ratio of ^14^C activity/protein after 10 min of H_2_O_2_ treatment than in untreated cells are labeled. (B) Time course of the oxidation of proteins in E. coli wild-type cells upon treatment with H_2_O_2_. Details of the colored overlays of stained protein gels (shown in green) and phosphor images (shown in red) of cell extracts taken before (Co) and 2, 5, 10, and 30 min after addition of H_2_O_2_ to the cells. The selected proteins are FolE, Tpx, MetE, and TufB. Bar charts on the right show the oxidation-dependent change in ratio of ^14^C activity/protein of the protein spot labeled by an arrowhead. (C) Time course of the oxidation of MetE in E. coli wild-type cells upon treatment with 1 mM diamide. Details of the colored overlays of stained protein gels (shown in green) and autoradiographs taken on X-ray films (shown in red) of cell extracts taken before (Co) and 2, 10, and 30 min after addition of diamide to the cells.

To investigate thiol modification under conditions of exogenous oxidative stress, wild-type E. coli cells were grown to mid-logarithmic phase and exposed to oxidative stress treatment by addition of H_2_O_2_. Here, we focused on the 100 most heavily ^14^C-labeled proteins after 10 min of H_2_O_2_ treatment, and compared their ^14^C activity/protein ratio to the ratio immediately before and 2, 5, and 30 min after the addition of H_2_O_2_ (Figure 5; Table 4). From the 100 most thiol-modified proteins in oxidatively stressed E. coli cells, seven proteins showed increasing thiol modification upon exposure to oxidative stress. These included H_2_O_2_-detoxifying enzymes such as Tpx, as well as a number of biosynthetic enzymes such as MetE, GTP cyclohydrolase (FolE), and phosphoglycerate dehydrogenase (SerA).

**Table 4:**
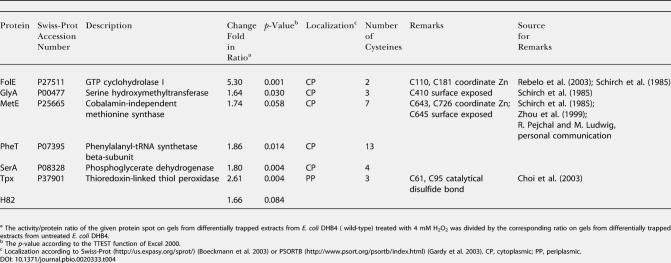
Oxidation Stress Sensitive Proteins in E. coli

^a^ The activity/protein ratio of the given protein spot on gels from differentially trapped extracts from E. coli DHB4 ( wild-type) treated with 4 mM H_2_O_2_ was divided by the corresponding ratio on gels from differentially trapped extracts from untreated E. coli DHB4

^b^ The *p*-value according to the TTEST function of Excel 2000

^c^ Localization according to Swiss-Prot (http://us.expasy.org/sprot/) (Boeckmann et al. 2003) or PSORTB (http://www.psort.org/psortb/index.html) (Gardy et al. 2003). CP, cytoplasmic; PP, periplasmic

Importantly, in the companion paper by Hondorp and Matthews (2004), MetE has been shown to be redox-regulated in vivo and in vitro. The surface-exposed cysteine 645 in MetE was found to be particularly sensitive to oxidative stress–induced thiol modifications. The authors showed that this thiol modification transiently inactivated the enzyme, which provided an excellent explanation for the observed methionine auxotrophy that accompanies oxidative stress in E. coli. To compare the kinetics and extent of our H_2_O_2_-induced thiol modification of MetE with the diamide-induced glutathionylation observed by Hondorp and Matthews, we analyzed the time course of MetE modification upon exposure of E. coli cells to 1 mM diamide. As shown in Figure 5C, MetE was maximally thiol-modified within 2 min of diamide treatment and maintained its high level of thiol modification over at least 30 min of incubation. This was in excellent agreement with the in vivo thiol trapping conducted by Hondorp and Matthews, who showed that at their first time point of 15 min, all of MetE was in the oxidized state.

The second metabolic enzyme that we identified as being particularly sensitive to oxidative stress was FolE, which catalyzes the committed step in the synthesis of the one-carbon donor tetrahydrofolate. Analysis of its crystal structure revealed that FolE contains a cysteine-coordinating zinc center, which escaped prior detection because of its high oxidation sensitivity in vitro (Rebelo et al. 2003). Air-oxidation of FolE leads to the inactivation of the enzyme. We have now observed that FolE is one of the major targets of H_2_O_2_ treatment in *E. coli,* with a more than 5-fold increase in ^14^C activity/protein ratio upon H_2_O_2_ treatment. This suggested that FolE is also transiently oxidized and inactivated upon oxidative stress in vivo, a finding that would clearly make physiological sense. Tetrahydrofolate is a highly oxidation-sensitive compound, and synthesizing it under oxidative stress conditions would be extremely wasteful for the cell.

Analysis of the time course of thiol modification in FolE during H_2_O_2_-induced oxidative stress treatment showed a steady increase in thiol modification. This was in contrast to the time course of thioredoxin substrate proteins such as Tpx, whose thiol modification peaked around 2–5 min after the start of the oxidative stress treatment (Figure 5B). This suggests that FolE is indeed a protein whose redox state is not controlled by thioredoxin and that is especially sensitive to oxidative stress treatment.

A large number of cysteine-containing cytoplasmic proteins (e.g., isocitrate dehydrogenase), as well as all of the identified periplasmic proteins, did not show any significant increase in oxidation-induced thiol modification. This indicated that under oxidative stress conditions, the majority of cytosolic proteins remain reduced and confirmed that the periplasmic proteins were already fully oxidized under aerobic growth conditions. The probably best-known redox-regulated proteins in *E. coli,* the oxidative stress transcription factor OxyR and the molecular chaperone Hsp33, were not among the 100 most thiol-modified proteins after 10 min of oxidative stress. This was not very surprising, given that OxyR is a low-abundance protein and Hsp33 has an extremely low pI (pH 4.35) and cannot be detected by our 2D gel system. The fact, however, that we identified a number of proteins that either have been shown (Tpx and MetE) or were predicted (FolE) to undergo thiol modification upon oxidative stress in vivo, made us very confident that thiol modifications play regulatory or functional roles in the other proteins that we discovered as well (e.g., GlyA, PheT, and SerA). All of these proteins have numerous cysteine residues, which are either surface exposed or might play other functional roles that have not yet been identified (Table 4). Detailed biochemical analysis is now required to investigate the exact role that thiol modifications play in these potentially redox-regulated proteins.

### Conclusion: A Widely Applicable New Method to Visualize Thiol Modifications In Vivo

Over the past few years, a variety of reversible oxidative cysteine modifications have been discovered that regulate the activity of eu- and prokaryotic proteins. The most prominent modification is disulfide bond formation, but also transient glutathionylation of cysteines or oxidation to sulfenic acid have been found to play an important regulatory role in many proteins (Barrett et al. 1999; Kim et al. 2002). These modifications are usually transiently introduced by specific stress conditions such as peroxide or disulfide stress and are resolved by cellular thiol-disulfide oxidoreductases such as thioredoxin and glutaredoxin, which are usually upregulated during these stress conditions (Potamitou et al. 2002).

Several new proteomic strategies have now been developed to probe for potential substrate proteins of cellular thiol-disulfide oxidoreductases and proteins that undergo thiol modifications during stress conditions. These strategies involve the use of radioactive glutathione to detect glutathionylated proteins under oxidative stress conditions (Fratelli et al. 2002), fluorescent thiol-reactive dyes to identify thioredoxin-targeted proteins (Yano et al. 2001), or active-site mutants of thiol-disulfide oxidoreductases, whose failure to complete the enzymatic reaction leads to an irreversible disulfide crosslink between thiol-disulfide oxidoreductase and substrate, which can then be identified by SDS-PAGE or diagonal PAGE in combination with mass spectrometry (Motohashi et al. 2001; Balmer et al. 2003; Kadokura et al. 2004). Very recently a study also examined proteins that interact very tightly with tap-tagged thioredoxin in E. coli (Kumar et al. 2004). This technique, however, was unable to distinguish between proteins that require thiol-disulfide exchange reactions with thioredoxins (i.e., enzymatic substrates) and proteins that simply associate with thioredoxin without the involvement of thiol chemistry. This was especially emphasized by the finding that at least 25% of the thioredoxin-associated proteins identified by Kumar et al. did not contain any cysteines.

With the help of these various methods, a number of proteins have been identified that serve as substrate proteins of different thiol-disulfide oxidoreductases in E. coli and other organisms. We have now developed a technique that allows us to globally monitor and compare the thiol-disulfide status of all cellular proteins that can be resolved in 2D gels. With this technique, the substrate proteins of all major oxidoreductase systems can be identified and dissected simply by comparing the thiol status of proteins in the appropriate mutant strains. In prokaryotes, for instance, substrate overlap and differences between the thioredoxin A and C can be analyzed by simply comparing the thiol disulfide status in *trxA^−^* and *trxC^−^* cells. The substrate specificity of the complete thioredoxin system can then be distinguished from the substrate specificity of the glutaredoxin systems by comparing the thiol-disulfide status of cellular proteins in strains lacking one of the systems altogether.

Most importantly, this technique should be widely applicable to many different cell types and organisms. Preliminary experiments in yeast, for instance, showed that AdhE, GapDH, and SdhB are major targets for oxidative thiol modifications in yeast (data not shown). We found the respective E. coli homologs to be among the most redox-sensitive proteins in strains lacking thioredoxin A. The reason why this method is applicable to both pro- and eukaryotic cells is the rapid thiol-quenching step that involves the incubation and lysis of cells in the presence of TCA. This immediately stops all thiol-disulfide exchange reactions. All subsequent trapping steps are then conducted with the soluble proteins under denaturating conditions. These unique features should allow us and others to monitor and visualize the in vivo thiol status of cellular proteins upon exposure of various cells and organisms to virtually every physiological or pathological condition that is accompanied by oxidative stress.

## Materials and Methods

### 

#### Bacterial strains.

E. coli DHB4 (F′ *lac*-*pro lacI* ^Q^/Δ*(ara*-*leu)7697 araD139* Δ*lacX74 galE galK rpsL phoR* Δ*(phoA)*PvuII Δ*malF3 thi*) (herein referred to as wild-type), WP570 (DHB4 Δ*trxA*) (Prinz et al. 1997), and LL029 (DHB4 *dsbA*::kan) were grown aerobically in glucose MOPS minimal medium (Neidhardt et al. 1974) containing 40 μg/ml L-leucine and 10 μM thiamine at 37 °C. Oxygen-limited cultures were grown in completely full 15-ml screw cap tubes. E. coli LL029 was obtained by P1 transduction of a *dsbA*::kan insertion mutation into DHB4. The *dsbA*-null strain AH55 was used as the source of the P1 transduction (Hiniker and Bardwell 2004).

#### Harvest of cell samples.

Wild-type E. coli and the respective mutant cells were grown to an OD_600_ of 0.4 at 37 ^o^C. To expose wild-type E. coli cells to oxidative stress treatment, the cells were then treated with 4 mM H_2_O_2_ or 1 mM diamide for the duration indicated. Then 1.8 ml of the cell culture was harvested directly into 200 μl of ice-cold 100% (w/v) TCA and stored on ice for at least 20 min.

#### Differential thiol trapping of cellular proteins.

The TCA-treated cells were centrifuged (13,000*g*, 4 °C, 30 min), and the resulting pellet was washed with 500 μl of ice-cold 10% (w/v) TCA followed by a wash with 200 μl of ice-cold 5% (w/v) TCA. The supernatant was removed completely, and the pellet was resuspended in 40 μl of denaturing buffer (6 M Urea, 200 mM Tris-HCl (pH 8.5), 10 mM EDTA, and 0.5 % [w/v] SDS) supplemented with 100 mM IAM. This first alkylation procedure irreversibly modified all free thiol groups that were made accessible by the urea and SDS-denaturation of the proteins. After 10 min of incubation at 25 °C, the reaction was stopped by adding 40 μl of ice-cold 20% (w/v) TCA. After 20 min of incubation on ice, the alkylated proteins were centrifuged again, and the pellet was washed with TCA as described before. The protein pellet was then dissolved in 20 μl of 10 mM DTT in denaturing buffer to reduce all reversible thiol modifications such as disulfide bonds and sulfenic acids. After a 1-h incubation at 25 °C, 20 μl of a solution of 100 mM radioactively labeled [^14^C-1]-IAM in denaturing buffer was added to titrate out the DTT and to irreversibly alkylate all newly reduced cysteines. The reaction mixture was incubated for 10 min at 25 °C. The reaction was stopped by adding 40 μl of 20% (w/v) TCA. After precipitation on ice and subsequent centrifugation, the pellet was washed first with TCA and then three times with 500 μl of ice-cold ethanol (for schematic overview see Figure 1). Reverse-trapping experiments were conducted as described except that the first alkylation procedure was performed with [^14^C-1]-IAM while the second alkylation step was performed with unlabeled IAM. For protein identification purposes, thiol-trapping experiments using nonradioactive IAM in both alkylation steps were performed in parallel.

#### 2D gel electrophoresis.

The pellet of the thiol-trapped proteins was dissolved in 500 μl of rehydration buffer (7 M urea, 2 M thiourea, 1% [w/v] Serdolit MB-1, 1% [w/v] dithiothreitol, 4% [w/v] Chaps, and 0.5% [v/v] Pharmalyte 3–10), and the 2D gel electrophoresis was performed as previously described (Hiniker and Bardwell 2004).

#### Staining of the gels, storage phosphor autoradiography, and image analysis.

Gels were stained using colloidal Coomassie blue stain (Neuhoff et al. 1985) and scanned using an Expression 1680 scanner with transparency unit (Epson America, Long Beach, California, United States) at 200-dpi resolution/16-bit grayscale. Phosphor images were obtained by exposing LE Storage Phosphor Screens (Amersham Biosciences, Piscataway, New Jersey, United States) to dried gels for 7 d. The phosphor image screens were read out with the Personal Molecular Imager FX (Biorad, Hercules, California, United States) at a resolution of 100 μm. The original image size of the phosphor image was changed to a resolution of 200 dpi with PhotoShop 7.0 (Adobe Systems, San Jose, California, United States). The phosphor images and images of the stained proteins were analyzed using Delta 2D Software (Decodon, Greifswald, Germany).

#### Data analysis.

For each of the described experiments, at least four individually trapped samples of cultures were obtained from at least two independent cell cultures. The only exceptions were the time course of H_2_O_2_ treatment at the time points 2, 5, and 30 min, the DsbA experiments under oxygen limitation, and the time course of diamide treatment. For each of the experiments, the phosphor image with the highest overall ^14^C activity was chosen for spot detection. The 100 most abundant spots were chosen from the detected set of spots and the boundaries transferred to all other phosphor images and protein gel images using the Delta 2D “transfer spots” function. The absolute intensity for each of these 100 spots on the protein gels and the phosphor image was determined to quantitatively describe the amount of protein and ^14^C activity for each protein spot. These absolute spot intensities were then normalized over all 100 spots (for trapping and reverse-trapping of wild-type cells, and for all H_2_O_2_ experiments). This normalization scheme was changed when the thiol-disulfide status of the *dsbA* mutant strain was analyzed. This was based on the consideration that a large number of the most intense spots on the phosphor images are heavily thiol-modified periplasmic proteins, which are putative DsbA substrate proteins. Normalizing over those protein spots would largely affect our data analysis. We therefore decided to normalize over four of the most abundant intracellular spots, TufB isoform (IF) 1, GapA IF 1, AhpC, and GroEL, whose thiol-disulfide status was not affected by the absence or presence of DsbA. In the case of the *trxA* mutant strain, similar considerations led us to normalize over four of the most abundant periplasmic protein spots, OmpA IF 1, OmpA IF 2, HisJ, and ArtJ, whose thiol-disulfide status was not influenced by the lack of TrxA activity. Finally, the ratio of ^14^C activity/protein was calculated by dividing the normalized intensity of the protein spot on the phosphor image by the corresponding normalized intensity of the Coomassie blue–stained protein spot. For a protein to be considered significantly thiol-modified, the average of the ^14^C activity/protein ratio for a given protein spot had to be at least 1.5-fold above the average of the ^14^C activity/protein ratio of this protein under control conditions.

#### Identification of proteins from 2D gels.

Thiol-trapped samples using nonradioactive IAM in both alkylation steps were separated on 2D gels and used to excise proteins of interest. These proteins were identified by Peptide Mass Fingerprinting at the Michigan Proteome Consortium (http://www.proteomeconsortium.org).

## Supporting Information

### Accession Numbers

The Swiss-Prot (http://www.ebi.ac.uk/swissprot/) accession numbers for the gene products discussed in this paper are 30S ribosomal subunit protein S2 (P02351), 50S ribosomal subunit protein L5 (P02389), AceF (P06959), AcnB (P36683), AdhE (P17547), AhpC (P26427), and γ-glutamyl phosphate reductase (P07004), ArtI (P30859), ArtJ (P30860), aspartate semialdehyde dehydrogenase (P00353), carbonic anhydrase (P36857), carbonic anhydrase (P36857), citrate synthase (P00891), DAHP synthetase (P00886), DppA (P23847), DsbA (P24991), GapA (P06977), glutamyl-tRNA synthetase (P04805), GroEL (P06139), GTP cyclohydrolase I (P27511), HisJ (P39182), Hsp33 (P45803), Imp (P31554), isocitrate dehydrogenase (P08200), Lpd (P00391), MetE (P25665), MetQ/YaeC (P28635), NusA (P03003), OmpA (P02934), OppA (P23843), OxyR (P11721), phenylalanyl-tRNA synthetase beta-subunit (P07395), phosphate import ATP-binding protein (P07655), phosphoribosylaminoimidazole synthetase (P08178), phosphoribosylaminoimidazole-succinocarboxamide synthetase (P21155), phosphotransferase system enzyme I (P08839), PhoU (P07656), porin protein E (P02932), P-specific transport protein (P06128), pyruvate kinase I (P14178), SdhB (P07014), SerA (P08328), serine hydroxymethyltransferase (P00477), SspA (P05838), succinyl-CoA synthetase (P07459), Tpx (P37901), trigger factor (P22257), TufB (P02990), and UgpB (P10904).

## Abbreviations

2D: two-dimensional
GapDH: glyceraldehyde-3-phosphate dehydrogenase
H_2_O_2_: hydrogen peroxide
IAM: iodoacetamide
IF: isoform
TCA: trichloracetic acid
